# Prevalência da Infecção pelo *Trypanosoma cruzi* em Doadores de Sangue

**DOI:** 10.36660/abc.20190285

**Published:** 2020-12-01

**Authors:** Alanna Carla da Costa, Eduardo Arrais Rocha, José Damião da Silva, Arduina Sofia Ortet de Barros Vasconcelos Fidalgo, Francisca Mylena Melgaço Nunes, Carlos Eduardo Menezes Viana, Vânia Barreto Aguiar F. Gomes, Maria de Fátima Oliveira

**Affiliations:** 1 Universidade Federal do Ceará FortalezaCE Brasil Universidade Federal do Ceará, Fortaleza, CE - Brasil; 2 Centro de Hematologia e Hemoterapia do Ceará FortalezaCE Brasil Centro de Hematologia e Hemoterapia do Ceará, Fortaleza, CE – Brasil

**Keywords:** Doença de Chagas/complicações, Doença de Chagas/epidemiologia, Trypanosoma Cruzi, Banco de Sangue, Testes Sorológicos

## Abstract

**Fundamento:**

A doença de Chagas (DC) é considerada um problema de saúde pública na América Latina. A região nordeste, principalmente o estado do Ceará, ainda representa grande preocupação em termos de risco de transmissão da doença.

**Objetivo:**

Estimar a prevalência de T. cruzi em doadores de sangue do estado do Ceará.

**Métodos:**

Trata-se um de estudo retrospectivo descritivo realizado no período de 2010 a 2015, a partir de dados registrados no sistema informatizado do Centro de Hematologia e Hemoterapia do Ceará (HEMOCE).

**Resultados:**

Dos 763.731 potenciais doadores de sangue, 14.159 foram considerados impedidos de fazer a doação devido à sorologia, sendo que 1.982 (0,33%) o foram devido à positividade/inconclusão para doença de Chagas. Compareceram à Hemorrede para a repetição 425 indivíduos, sendo confirmados 28,2% (120/425) como impedidos de doar devido a DC.

**Conclusão:**

Não houve redução significativa das sorologias positivas/inconclusivas no período entre 2010-2015, porém foi observada redução em relação a 1996/1997 no estado. A determinação da prevalência da doença de Chagas em bancos de sangue pode ser relevante como indicador do risco de transmissão transfusional em determinada região. Novos testes sorológicos para triagem com melhor acurácia são necessários, reduzindo o descarte desnecessário de bolsas de sangue, os custos para o Sistema Único de Saúde e a insegurança para os pacientes e familiares. (Arq Bras Cardiol. 2020; 115(6):1082-1091)

## Introdução

A doença de Chagas (DC) é considerada um problema de saúde pública na América Latina. Essa doença era exclusiva das Américas, mas, nas últimas décadas, se espalhou para outros continentes, devido à internalização da doença em virtude da migração de pessoas oriundas das áreas endêmicas para esses locais.^1^ No Brasil, a estimativa é de 2 a 3 milhões de pessoas infectadas.^2^

Na região nordeste, o Ceará é um dos estados em que ainda existe grande preocupação em termos de risco de transmissão da DC. Essa preocupação deve-se a três fatores: a região ainda permanece socialmente muito carente, possuí os mais altos índices de moradias propícias à colonização de triatomíneos, além do baixo nível operacional do Programa de Controle da Doença de Chagas (PCDCh) em todo o Brasil.^3 , 4^

Com relação à transmissão da DC, as duas formas de maior importância epidemiológica são a vetorial e a transfusional.^2 , 5 , 6^ A partir de 1940, a prática da transfusão de sangue se generalizou por toda a América Latina, o que contribuiu para aumentar o risco de doença de Chagas transfusional.^7 - 12^ Diante disso, a transfusão de sangue começou a ter importância epidemiológica a partir de 1944 e passou a ser tecnicamente avaliada em 1951. Em 1969, foi instituída a obrigatoriedade da triagem sorológica para doença de Chagas nos bancos de sangue do Brasil. Essa ação foi estabelecida pelo Ministério da Saúde com o fim de controlar e aumentar a segurança das doações realizadas.^13^

A portaria nº 158, de 04 de fevereiro de 2016 do Ministério da Saúde, considera doador inapto aquele que teve contato domiciliar com triatomíneos em área endêmica, e aqueles com diagnóstico clínico ou laboratorial para doença de Chagas. Além disso, no Art. 130 da mesma portaria, considera-se obrigatória a realização de teste sorológico de alta sensibilidade para DC, em cada doação realizada.^14^

O objetivo do estudo é estimar a prevalência de *T. cruzi* em doadores de banco de sangue do estado do Ceará, visto ser pouco explorado. Os resultados do presente estudo servirão de alerta para os membros da vigilância epidemiológica, para que estabeleçam medidas de prevenção, tratamento e acompanhamento dos indivíduos infectados pelo *Trypanosoma cruzi* e para os indivíduos que vivem em áreas de risco.

## Metodologia

### Desenho do estudo, local da pesquisa e amostra

**Figura 1:**
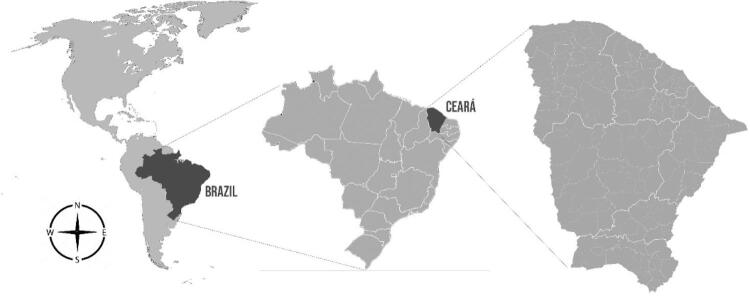
– Mapas mostrando a localização do estudo. Fonte: Elaborada pelo próprio autor.

Trata-se um de estudo retrospectivo descritivo que foi realizado a partir de dados registrados no sistema informatizado do Centro de Hematologia e Hemoterapia do Ceará (HEMOCE), de todos os potenciais doadores de sangue da Hemorrede Pública Estadual do período de 2010 a 2015 ( Figura 1 ). Os dados pessoais dos doadores foram preservados, e estes foram identificados por número de registo para garantir a confidencialidade.

A Hemorrede Pública Estadual é formada por um Hemocentro Coordenador, com sede em Fortaleza; quatro Hemocentros Regionais, localizados nos municípios de Sobral, Quixadá, Crato e Iguatu; um Hemonúcleo, em Juazeiro do Norte; um Posto de Coleta de Sangue no Instituto Dr. Jose Frota – IJF, e sessenta e quatro Agências Transfusionais localizadas nos hospitais atendidos pela Hemorrede em Fortaleza e municípios do interior do Ceará. Cada hemocentro é responsável pela realização do atendimento a doadores e pacientes em sua área de cobertura, tendo competência para realizar todos os passos do ciclo do sangue, à exceção da sorologia que está centralizada há mais de uma década no Hemocentro de Fortaleza, onde foram coletados os dados referentes aos doadores de sangue.

A triagem sorológica para doença de Chagas no período do estudo foi realizada pela técnica de quimioluminescência que consiste na interação de anticorpos presentes no soro de pacientes infectados com os epítopos antigênicos presentes na placa que, após incubação de antigamaglobulina biotinilada e streptoavidina conjugada com enzima, na presença do reagente luminol, são capazes de emitir luz.^15 , 16^

Se o resultado for positivo ou inconclusivo, o teste é repetido em duplicata na mesma amostra. Caso o resultado permaneça positivo ou inconclusivo, o paciente é recrutado ao serviço para uma segunda coleta de sangue. Após a coleta é realizado novamente o teste de triagem (quimioluminescência), e, caso o resultado seja positivo ou inconclusivo na repetição do teste, será realizado o teste confirmatório e o doador já é recusado definitivamente, mesmo que o teste confirmatório seja negativo. O teste confirmatório dever ser um teste de elevada especificidade, o Hemoce utiliza imunofluorescência indireta (IFI) ou *western blot,* ^15^ dependendo dos processos licitatórios, já que se trata de um órgão público.

A IFI consiste na detecção de anticorpos presentes no soro de infectados que, quando incubados sobre uma lâmina com antígenos fixados de *T. cruzi* , ligam-se, formando o complexo antígeno-anticorpo, e que são revelados por uma antigamaglobulina humana marcada com isotiocianato de fluoresceína. O teste de *western blotting* consiste em um método em biologia molecular e bioquímica para detectar proteínas em um homogenato ou um extrato de um tecido biológico. Essa técnica usa eletroforese em gel para separar as proteínas desnaturadas por massa. As proteínas são então transferidas do gel para uma membrana de nitrocelulose, onde são usados como sondas, anticorpos específicos à proteína.^15^

**Figura 2:**
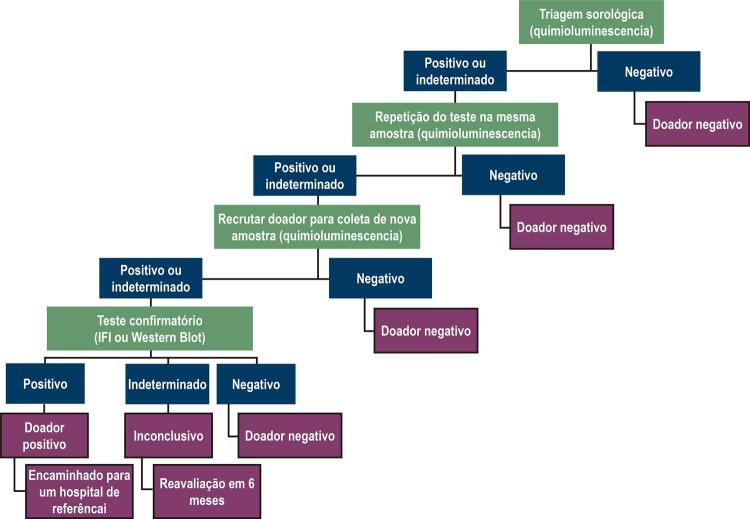
– Fluxograma da Triagem de Potencial doador de sangue para doença de Chagas na Hemorrede Pública Estadual em 2017. IFI: imunofluorescência Indireta.

Caso o resultado seja positivo ou inconclusivo no teste confirmatório, o paciente é encaminhado para a clínica médica do Hospital Universitário Walter Cantídio ou Hospital de Messejana, que são unidades de referência para o acompanhamento clínico dos indivíduos com doença de Chagas crônica no estado do Ceará ( Figura 2 ).

### Análise dos dados

A ocorrência de doadores inaptos por doença de Chagas foi calculada, e uma análise estatística descritiva foi realizada a partir de frequências absolutas e relativas. Para a caracterização epidemiológica dos indivíduos impedidos de doar devido a DC confirmada, os seguintes parâmetros foram considerados: gênero (masculino e feminino), faixa etária (18 - 29 anos e acima de 30 anos), ocupação, naturalidade e procedência (Fortaleza ou interior do estado). A análise descritiva foi realizada no programa Microsoft Excel versão 2013.

### Aspectos éticos

O presente estudo foi aprovado pelo Comitê de Ética em Pesquisa da Universidade Federal do Ceará (COMEPE-UFC) sob o parecer de número 1.482.674, CAAE: 53833816.5.0000.5054 que foi julgado de acordo com as normas que regulamentam a pesquisa com seres humanos do Conselho Nacional de Saúde (Resolução CNS 466/12).

## Resultados

Durante o período do estudo (janeiro de 2010 a dezembro de 2015), a Hemorrede Pública Estadual do Ceará recebeu um total de 763.731 potenciais doadores de sangue. Destes, 155.378 (20,3%) foram excluídos na triagem clínica, e entre os motivos da exclusão estão anemia, hipertensão, alcoolismo, uso de drogas, comportamento de risco para doenças sexualmente transmissíveis, hepatite, malária, doença de Chagas e outras causas. Foram recusados na triagem clínica devido doença de Chagas/picada de triatomíneo 38 (0,02%) potenciais doadores, sendo 21(55,2%) homens e 17(44,8%) mulheres. Logo, realizaram a pesquisa de anticorpos contra *T. cruzi* (Triagem sorológica) 608.353 (79,6%) indivíduos.

No período do estudo, foram descartadas 14.159 bolsas de sangue, porque foram consideradas inadequadas para doação devido à sorologia positiva para alguma doença rastreada em banco de sangue, representando 2,32% do total de bolsas coletadas no período. Dos 608.353 candidatos a doação aprovados na triagem clínica, 1.982 (0,32%) foram considerados inaptos para doação devido à sorologia positiva/inconclusiva para doença de Chagas por meio do teste de quimioluminescência. Destes, 602 (30,37%) foram positivos e 1.380 (69,62%) foram inconclusivos ( Tabela 1 ). Os resultados inconclusivos corresponderam a 9,75% (1.380) do descarte de bolsas.

**Tabela 1:** – Total de doadores inaptos na triagem sorológica devido doença de Chagas na Hemorrede Pública Estadual de 2010 a 2015

ANO	DOADORES*	POS (N)	POS (%)	IND (N)	IND (%)	TOTAL INAPTOS (N)	TOTAL INAPTOS (%)
2010	94847	86	0,09	244	0,25	330	0,34
2011	100019	89	0,09	231	0,24	320	0,33
2012	98815	89	0,09	374	0,38	463	0,47
2013	99037	114	0,12	365	0,37	479	0,49
2014	105281	112	0,11	84	0,08	196	0,19
2015	110354	112	0,1	82	0,07	194	0,17
**TOTAL**	**608353**	**602**	**0,1**	**1380**	**0,23**	**1982**	**0,33**

POS: positivos; IND: indeterminados. *Total de doadores que realizaram a triagem sorológica. Fonte: Hemorrede Pública Estadual do Ceará (2010 – 2015).

Os resultados mostraram um aumento de 16,35% no número de potenciais doadores quando comparado 2010 a 2015. Em relação à prevalência por ano, houve uma diminuição de 50% (0,34% em 2010, 0,17% em 2015).

**Figura 3:**
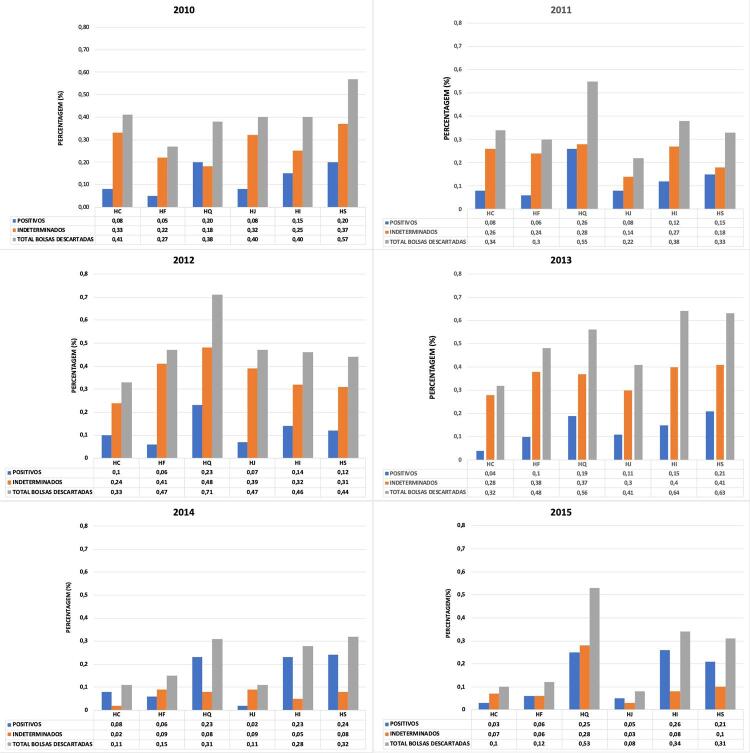
– Prevalência da Doença de Chagas nos Hemocentros da Hemorrede Pública Estadual no período de 2010 a 2015. Fonte: Elaborada pelo próprio autor.

Para verificar se a doença de Chagas ainda está presente em áreas historicamente endêmicas, foram analisadas as prevalências nos hemocentros de Sobral, Quixadá e Iguatu, considerando resultados positivos e inconclusivos. O hemocentro de Sobral apresentou maior prevalência para doença de Chagas no ano de 2010, 0,57% (78/13674). No ano de 2011, o hemocentro de Quixadá também apresentou 0,55% (29/5312), e no ano de 2012 esse mesmo hemocentro apresentou 0,71% (43/6075) e em 2013 0,56% (33/5914) ( Figura 3 ).

Os hemocentros de Quixadá, Sobral e Iguatu apresentaram os maiores índices de soroprevalência de infecção por *T. cruzi* no período estudado 0,51% (171/33413), 0,42%(356/85150) e 0,40% (140/35286), respectivamente.

Os anos de 2012 e 2013 apresentaram as maiores porcentagens de doadores inaptos devido a doença de Chagas, 0,47 e 0,49% respectivamente, com uma subsequente diminuição nos anos seguintes 2014 e 2015, com os menores valores registados 0,19 e 0,17% ( Tabela 1 ). Dos doadores inaptos devido a DC, 847 (42,73%) já haviam doado sangue anteriormente no período estudado.

Dos 1.982 doadores convocados para repetição do teste (positivos e inconclusivos na triagem sorológica por doença de Chagas), 757 (38,19%) indivíduos não compareceram à Hemorrede para a repetição do teste. Logo, 1.225 (61,8%) indivíduos realizaram a repetição do teste.

Na repetição do teste de triagem por quimioluminescência, 92 indivíduos apresentaram resultado inconclusivo e 333, resultado positivo. Essas amostras reagentes foram submetidas ao teste confirmatório (IFI ou *western blot* ). Destes, 305 indivíduos apresentaram teste confirmatório negativo, 48, resultados inconclusivos e 72, resultados positivos ( Tabela 2 ). Logo, foram confirmados 120 indivíduos (28,23%) inaptos para doação devido a doença de Chagas.

**Tabela 2:** – Doadores de sangue considerados inaptos na triagem sorológica e recrutados para a repetição do teste e realização do teste confirmatório de 2010 a 2015 (n: 1982)

REPETIÇÃO DO TESTE DE TRIAGEM (Quimioluminescência)						TESTE CONFIRMATÓRIO (IFI ou *Western Blo* t)			
ANO	NÃO RETORNARAM	REPETIÇÃO NEG	REPETIÇÃO IND	REPETIÇÃO POS	TOTAL	CONF. NEG	CONF. IND	CONF. POS	TOTAL
2010	103	142	11	26	282	21	8	10	39
2011	124	126	13	47	310	49	11	11	71
2012	145	131	21	101	398	102	10	9	121
2013	160	235	18	54	467	41	1	6	48
2014	105	80	20	39	244	45	7	19	71
2015	120	86	9	66	281	47	11	17	75
**TOTAL**	**757**	**800**	**92**	**333**	**1.982**	**305**	**48**	**72**	**425**

NEG: negativo; IND: indeterminado; POS: positivo; CONF: confirmatório. IFI: imunofluorescência Indireta. Fonte: Hemorrede Pública Estadual do Ceará (2010 – 2015).

Desses indivíduos inaptos confirmados devido a doença de Chagas (n=120), 78 (65%) eram do sexo masculino, 100 (83,3%) tinham idade maior que 30 anos, 100 (83,3%) eram doadores de primeira vez, e 20 (16,7%) já haviam doado sangue anteriormente. Possuíam o ensino médio completo 45 (37,5%), e 32 (26,7%), o ensino fundamental incompleto, enquanto 88 (73,3%) eram naturais de cidades do interior do estado do Ceará, e 62 (51,7%) eram procedentes da capital Fortaleza ( Tabela 3 ).

**Tabela 3:** – Características sociodemográficas dos doadores considerados inaptos com confirmação de doença de Chagas na Hemorrede Pública Estadual de 2010 a 2015

	Positivos		Indeterminados		Total de inaptos	
Variáveis	n	%	n	%	n	%
**Sexo**						
Masculino	43	59,7	35	73	78	65
Feminino	29	40,3	13	27	42	35
Total	72	100	48	100	120	100
**Idade **	**n**	**%**	**n**	**%**	**n**	**%**
≤ 30 anos	8	11,1	12	25	20	16,7
>30 anos	64	88,9	36	75	100	83,3
Total	72	100	48	100	120	100
**N de doações realizadas**	**n**	**%**	**n**	**%**	**n**	**%**
0	69	95,8	31	64,6	100	83,3
≥ 1	3	4,2	17	35,4	20	16,7
Total	72	100	48	100	120	100
**Escolaridade**	**n**	**%**	**n**	**%**	**n**	**%**
Analfabeto	4	5,5	1	2,1	5	4,2
Fund. Incompleto	21	29,2	11	23	32	26,7
Fund. Completo	8	11,1	3	6,2	11	9,2
Médio Incompleto	10	13,9	1	2,1	11	9,2
Médio Completo	24	33,3	21	43,7	45	37,5
Superior Incompleto	0	0	5	10,4	5	4,2
Superior Completo	2	2,8	5	10,4	7	5,8
Não informado	3	4,2	1	2,1	4	3,3
Total	72	100	48	100	120	100
**Naturalidade**	**n**	**%**	**n**	**%**	**n**	**%**
Fortaleza	7	9,7	15	31,2	22	18,3
Região Metropolitana	3	4,2	4	8,3	7	5,8
Interior do estado	61	84,7	27	56,3	88	73,3
NI	1	1,4	2	4,2	3	2,5
Total	72	100	48	100	120	100
**Procedência**	**n**	**%**	**n**	**%**	**n**	**%**
Fortaleza	30	41,7	32	66,6	62	51,7
Região Metropolitana	16	22,2	6	12,5	22	18,3
Interior do Estado	25	34,7	10	21	35	29,2
NI	1	1,4	0	0	1	0,8
Total	72	100	48	100	120	100

FUND: fundamental; NI: não informado. Fonte: Hemorrede Pública Estadual do Ceará (2010 – 2015).

## Discussão

A doença de Chagas, aos poucos, vem deixando de ter a relevância que teve historicamente no quadro sanitário brasileiro, mesmo assim ainda representa um desafio sob muitos aspectos. Fiusa-Lima & Silveira em 1984^16^ encontraram em seu estudo uma prevalência geral de 3,05% de infecção chagásica na região nordeste (Brasil = 4,40%). Alencar realizou os primeiros estudos sobre essa doença no Ceará em 1987 e detectou uma prevalência estimada de 14,8%, destacando o município de Limoeiro do Norte que apresentou 16,7%.^17^ Apesar da redução significativa da transmissão vetorial a partir da década 1990, a doença de Chagas é considerada, pela Organização Mundial de Saúde como “negligenciada”, o que a torna parte de uma agenda política e programática de controle, que deve ser seguida pelos países endêmicos.^18^

Do ponto de vista dos hemocentros, a doença permanece como objeto de vigilância contínua, visto que a população de doadores engloba número considerável de indivíduos que já viveram sob condições sanitárias e ambientais que favoreciam a transmissão da doença e alguns indivíduos que ainda vivem nessas condições. Assim, a realização de estudos epidemiológicos em serviços de hemoterapia se torna importante, não apenas pela relevância transfusional, mas também como opção para avaliar a transmissão da doença em determinados municípios.

No presente estudo, a prevalência da DC foi de 0,33% na triagem sorológica da Hemorrede Pública do Estado do Ceará. Resultados semelhantes foram encontrados em outras regiões do país, como no estudo realizado no Hemocentro da cidade de Ituiutaba, no Triângulo Mineiro, que detectou que 0,23% dos candidatos tinham sorologia positiva para a doença de Chagas e 0,27% apresentaram resultados sorológicos inconclusivo no período de 2001 a 2011.^19^ No período de 1995 a 2009 foi realizado um estudo no Centro Regional de Sangue de Uberaba (HRU) e foi encontrada uma prevalência sérica de 0,2% de impedimento devido à doença de Chagas.^20^

Uma revisão bibliográfica realizada por Costa et al.^21^ analisou a inaptidão de candidatos à doação de sangue relacionada à soropositividade para doença de Chagas nas diferentes regiões do Brasil (nordeste, sudeste, sul). Dessa forma, as maiores prevalências de doadores inaptos com sorologia positiva para doença de Chagas ocorreram na região Nordeste, especificamente na cidade de Iguatu – CE, e na região Sudeste, na cidade de Patos de Minas - MG, com percentuais de 1,90% e 1,20% respectivamente. Na região Sul do país os percentuais de prevalência para doadores inaptos positivos para a DC variaram entre 0,40% e 0,47% em Porto Alegre e Pelotas, respectivamente.

No presente estudo observamos que o hemocentro de Iguatu apresentou uma das maiores prevalências do período estudado (0,41%), porém, quando comparado com o estudo de Costa et al.^21^ observa-se uma redução na prevalência de inaptidão sorológica devido DC em Iguatu- CE nos anos de 2010 a 2015.

O hemocentro de Sobral apresentou uma das maiores prevalências do período estudado quando comparado com os outros hemocentros do estado ( Figura 3 ). Esse dado pode ser explicado pelo fato da cidade de Sobral ser considerada de alto risco para a doença de Chagas vetorial. Esse município apresenta um cenário eco-epidemiológico único caracterizado por focos de triatomíneos em assentamentos urbanos informais com moradias abaixo do padrão. Espécies de triatomíneos nativos como *Triatoma brasiliensis* e *Triatoma pseudomaculata* são vistas com frequência dentro das casas, estando muitas vezes infectados com *T. cruzi* . Esses focos têm o potencial de desenvolver casos agudos de doença de Chagas, nos mostrando que essa região ainda representa uma preocupação real para a população.^22^

Santana et al.^23^ analisaram a positividade para doença de Chagas entre doadores de sangue do Piauí entre 2004 e 2013. A prevalência de sorologia reagente para doenças de Chagas na triagem de doadores foi maior do que observada no presente estudo, em torno de 1%. Apenas 34,5% das amostras positivas na triagem foram encaminhadas para testes complementares. Nos testes confirmatórios, 84,4% apresentaram resultados negativos.^23^

No estado do Ceará, um estudo acerca da soroprevalência da infecção chagásica em bancos de sangue públicos foi o estudo de Silva et al.^24^ no período de 1996 a 1997, onde foram observados 34.943 doadores, dos quais, 377 (1,1%) apresentavam soropositividade para infecção chagásica.^24^ Quando comparamos com o presente estudo observamos que houve uma diminuição na inaptidão devido a DC na triagem sorológica que, até 2015, se encontrava em 0,33% ( Tabela 1 ). Isso representa, possivelmente, um reflexo das medidas para a melhoria da qualidade dos serviços de hemoterapia iniciadas desde os anos 80 em muitos estados brasileiros, que priorizaram a prática das doações de retorno com as fidelizadas e voluntárias. Além disso, como a transmissão nas áreas rurais vem diminuindo devido às medidas antivetoriais, era esperado que, com o passar do tempo, essa nova população de doadores apresentasse menores taxas de infecção.^24^

Além desses fatores, podemos ressaltar que as técnicas para detecção sorológica avançaram bastante, o que pode ter contribuído na diminuição dos resultados falso positivos.^25 - 27^

Diante do contexto, as políticas de controle da doença devem continuar existindo, para evitar surtos esporádicos como o que ocorreu em 1998 no estado do Ceará, devido a dificuldades na continuidade ao programa antivetorial (falta de inseticida e profissionais qualificados), o que provocou elevação no número de vetores capturados e alta frequência de sorologias reagentes entre os doadores.^28^

Deve-se alertar para o fato que a aplicação de inseticidas em domicílios não parece impedir a reintrodução contínua de espécimes de triatomíneos selvagens, e, como sabemos, o *Trypanosoma cruzi* circula dentro de um ciclo zoonótico doméstico, representando um desafio para as autoridades envolvidas no controle da doença de Chagas. Concentrar esforços das vigilâncias sanitárias nas três esferas de governo é necessário para erradicar essa doença e sua transmissão transfusional.^29 , 30^

Deve-se ressaltar que os serviços de hemoterapia não são obrigados por lei a realizar testes confirmatórios para qualquer uma das doenças detectadas pelos exames de rotina. Entretanto, conforme preconiza a Resolução RDC nº 343, de 13 de dezembro de 2002, assim como outras normas, qualquer doador com resultado sorológico não-negativo deve ser convocado para receber as orientações necessárias.^31^

Verificamos no presente estudo que muitos doadores não retornaram para a repetição do teste, e esse fato representa uma limitação do presente estudo, pois muitos potenciais doadores não realizaram os testes confirmatórios. O não retorno dos potenciais doadores representa perda de informações como a prevalência mais precisa da doença de Chagas em bancos de sangue.

A Hemorrede deve elaborar novas estratégias para recrutar esses indivíduos para a confirmação do diagnóstico e, no caso de resultado negativo, voltarem a ser doadores de sangue.^31^

Essa perda de informação é preocupante e pode ser decorrente de uma combinação de fatores, entre eles, a procedência de muitos doadores. Além da dificuldade de deslocamento do doador de procedência do interior para a capital, a carta de convocação é um instrumento que assusta, pois indica que o resultado do exame não foi normal, e muitos acabam não querendo saber o resultado de fato.^15^

Observou-se no presente estudo que a maioria dos doadores inaptos confirmados para DC (positivos/inconclusivo) era do sexo masculino, com idade superior a 30 anos, procedentes da capital Fortaleza, mas nascidos em cidades do interior do estado do Ceará. Embora outros estudos também mostrem essa predominância do sexo masculino nos indivíduos portadores da DC, não existe uma correlação positiva entre o sexo do doador e a sorologia reagente para a doença, pois ela afeta indistintamente homens e mulheres, mas pode ser justificada pelo fato que muitos homens trabalhavam em áreas rurais, tendo mais chances de entrar em contato com o triatomíneo, como também devido a diferenças culturais na prática da doação de sangue que comumente atribui aos homens o papel de doador por excelência.^28 , 32 - 34^

Quanto à naturalidade, sabe-se que muitos indivíduos sabidamente infectados moram ou moraram em regiões interioranas, que constituem os ecótopos naturais do inseto vetor e, posteriormente, muitos acabam migrando para os grandes centros urbanos como Fortaleza fugindo da seca e em busca de emprego e oportunidades.^35^ Apesar do certificado de erradicação da doença de Chagas por *Triatoma infestans* é importante deixar claro que essa espécie nunca foi encontrada no estado do Ceará, portanto o risco de transmissão da DC no Ceará é devido as espécies de triatomíneos mais prevalentes o *Triatoma brasiliensis* e o *Triatoma pseudomaculata.* Logo, o estado do Ceará ainda apresenta riscos de transmissão da doença.^3 , 35 - 37^

Além da forma vetorial, a forma oral de transmissão vem atingindo principalmente o norte e nordeste do país. No caso do homem, esta transmissão pode ocorrer de maneira esporádica, por meio da ingestão de alimentos contaminados com o parasito ou suas dejeções.^38^

Com relação à faixa etária, observou-se no período de estudo uma redução do número de indivíduos mais jovens infectados que pode ser avaliado como reflexo das medidas do controle vetorial no estado de Ceará. Com a redução geral da incidência da doença, torna-se cada vez menos frequente o ingresso de portadores da doença de Chagas no grupo etário de pessoas que doam sangue, em paralelo com a saída progressiva de infectados do rol de doadores por idade ou por morbidade devido à doença. Porém, é importante ressaltar que o aumento da realização de campanhas de doação de sangue gera uma maior demanda de pessoas de grupos etários bem variados, dificultando a identificação de uma diferença maior entre as faixas etárias analisadas.^39 , 40^

Paralelamente à diminuição de doadores de sangue portadores da doença de Chagas no estado do Ceará, vem chamando atenção a alta proporção de reações inconclusivas. Durante o período do presente estudo (2010-2015), observamos que na triagem sorológica 70,9% (1.380) dos resultados inaptos devido a DC foram inconclusivos, representando 5,99% do total de bolsas descartadas do período. Diversos estudos demonstraram que as reações inconclusivas representam frequentemente mais de 50% dos casos de inaptidão para doação devido à sorologia positiva para doença de Chagas em bancos de sangue, com essa taxa superior a 70% em alguns serviços.^41 , 42^

No Brasil, estima-se em 60% a ocorrência de reações inconclusivas nos três milhões de doações anuais, das quais 0,6% das bolsas coletadas são descartadas pela sorologia para *T. cruzi* , ou seja, 10.800 estão sendo descartadas por sorologia inconclusiva.^34^ É preocupante o número significativo de reações inconclusivas, já que, além dos custos acarretados pelo descarte de bolsas, existem as consequências que isso traz ao doador rotulado como portador de uma doença grave como a DC.

Discrepâncias nos resultados dos testes sorológicos ocorrem com certa frequência, às vezes duvidosas em um determinado teste e positivas ou mesmo negativas em outro. No presente estudo verificou-se que 847 indivíduos já haviam doado sangue anteriormente, e tais discrepâncias tornam-se mais evidentes e conflituosas em doadores de repetição, quando mais de uma dezena de reações sorológicas repetidamente negativas em doações prévias apresentam sorologia inconclusiva ou eventualmente positiva em doação subsequente.^43 - 45^

No presente estudo verificou-se que dos 1.982 indivíduos inaptos devido a DC, 1.225 (61,8%) retornaram para repetição do teste, e destes apenas 72 (5,8%) foram positivos no teste confirmatório (IFI ou *western blot* ) e 48 (3,9%) foram inconclusivos. A ocorrência de reações inconclusivas e reações falso-positivas nos testes de triagem sorológica traduz falhas na especificidade dos testes sorológicos, pois podem existir muitos indivíduos coinfectados com outras doenças, portanto, sensibilizados com outros antígenos, o que pode acarretar reações cruzadas nos testes sorológicos.

Consequentemente, muitos indivíduos sadios acabam sendo rotulados como portadores de uma doença que na maioria das vezes não possuem, levando a problemas psicológicos, sociais e econômicos ao doador excluído por ser erroneamente considerado chagásico, além de promover o descarte desnecessário de unidades de sangue nos hemocentros e importantes perdas financeiras para o Sistema Único de Saúde.^40^

Esses dados obtidos refletem as dificuldades na abordagem e na condução de doadores com reações sorológicas inconclusivas, que são quase todos não chagásicos, bem como a imprescindibilidade da implementação de medidas que permitam minimizar, ou mesmo eliminar, os resultados sorológicos duvidosos ou falso-positivos para a doença de Chagas nos testes de triagem sorológica.

Os testes confirmatórios apresentam boa sensibilidade e especificidade, porém os testes de triagem, como a quimioluminescência, apresentam elevada sensibilidade, e sabe-se que testes com alta sensibilidade têm sua especificidades comprometida, gerando resultados falso-positivos. Devido a esse fator, é importante a implementação de testes mais específicos na triagem sorológica de doadores de sangue.^28 , 30 , 31^

A determinação da prevalência da doença em bancos de sangue pode ser relevante como indicador do risco da doença de Chagas transfusional e do nível de transmissão da doença em uma determinada região, além de permitir avaliar indiretamente o programa de controle vetorial, fornecendo informações atuais sobre a doença no estado.

Dessa forma, é possível traçar estratégias locais que envolvam os esforços de todos os setores relacionados à área, como vigilância sanitária, hemocentros e laboratórios, unidos para eliminar a transmissão transfusional e melhorar a qualidade do sangue transfundido.

Além disso, os resultados obtidos alertam para a necessidade de introdução, nos hemocentros, de um método sorológico complementar que seja mais específico, a fim de minimizar o descarte desnecessário de bolsas de sangue e consequentemente indicar os valores reais da prevalência da doença em doadores de sangue. Logo, são necessários estudos que proponham novas medidas para a melhoria da acurácia dos testes sorológicos, o que, consequentemente, reduziria o descarte desnecessário de bolsas de sangue, diminuindo assim, os custos para o Sistema Único de Saúde.

## Conclusões

Dos potenciais doadores do período estudado, 1.982 considerados impedidos de fazer a doação devido sorologia positiva/inconclusiva para doença de Chagas. Foram confirmados como inaptos (positivos e inconclusivos) 28,2% (120/425) devido a DC. Não houve redução significativa das sorologias positivas/inconclusivas no período entre 2010-2015, porém foi observada redução em relação a 1996/1997 no estado. A determinação da prevalência da doença de Chagas em bancos de sangue pode ser relevante como indicador do risco de transmissão transfusional em determinada região. Novos testes sorológicos para triagem com melhor acurácia são necessários, reduzindo o descarte desnecessário de bolsas de sangue, os custos para o Sistema Único de Saúde, e a insegurança para os pacientes e familiares.
