# Supplementary material for: Prevalência da Infecção pelo Trypanosoma cruzi em Doadores de Sangue
Source: Arq Bras Cardiol. 2020 Dec 1;115(6):1082–91. [Article in Portuguese] doi: 10.36660/abc.20190285 (PMC8133715; doi:10.36660/abc.20190285)
Supplement: Translation

# Translation: Prevalence of Trypanosoma Cruzi Infection in Blood Donors

**Authors:** Alanna Carla da Costa, Eduardo Arrais Rocha, José Damião da Silva, Arduina Sofia Ortet de Barros Vasconcelos Fidalgo, Francisca Mylena Melgaço Nunes, Carlos Eduardo Menezes Viana, Vânia Barreto Aguiar F. Gomes, Maria de Fátima Oliveira

**Affiliations:** 1 Brazil Universidade Federal do Ceará, Fortaleza, CE - Brazil; 2 Brazil Centro de Hematologia e Hemoterapia do Ceará, Fortaleza, CE – Brazil

**Keywords:** Chagas Disease/complications, Chagas Disease/epidemiology, Trypanosoma Cruzi, Blood Banks, Sorologic Tests

## Abstract

**Background:**

Chagas disease (CD) is considered a public health problem in Latin America. The northeastern region, especially the state of Ceará, still represents a major concern in terms of the risk of transmission of CD.

**Objective:**

To estimate the prevalence of T. cruzi in blood donors from the state of Ceará.

**Methods:**

This is a retrospective descriptive study that was performed in the period from 2010 to 2015 from data recorded in the computerized system of the Hematology and Hemotherapy Center of Ceará (HEMOCE in Portuguese).

**Results:**

Of the 763,731 potential blood donors, 14,159 were serologically ineligible; 1,982 (0.33%) were serologically ineligible due to positive / inconclusive diagnosis for CD. A total of 425 individuals came to the HEMOCE to repeat the test, with 28.2% (120/425) declared ineligible for donations due to CD.

**Conclusion:**

No significant reduction of positive / inconclusive serology was observed in the period between 2010 and 2015, but a reduction was observed when compared to 1996/1997 in the state. The determination of the prevalence of CD in blood banks may be relevant as an indicator of the risk of CD transmission through blood transfusions in a given region. New serological tests for triage with better accuracy in screening are needed, in an attempt to reduce the unnecessary disposal of blood bags, reduce costs for the Brazilian Unified Health System, and diminish insecurity for the patient and family members. (Arq Bras Cardiol. 2020; 115(6):1082-1091)

## Introduction

Chagas disease (CD) is considered to be a public health problem in Latin America. This disease used to be exclusive to the Americas, but, in recent decades, it has spread to other continents, due to the internalization of the disease caused by the migration of people from endemic regions to these locations.^1^In Brazil, it is estimated that there are some two to three million infected people.^2^

In the northeast region of Brazil, the state of Ceará is one of the states in which there is still a great concern in terms of the transmission of CD. This concern is due to three main factors: the region is still, from a social point of view, quite needy. It also has the highest indices of homes conducive to the colonization of triatominae bugs, along with the low operational level of Chagas Disease Control Program (CDCP) throughout Brazil.^3 , 4^

As regards CD transmission, the two most important epidemiological forms are the vectorial and blood transfusions.^2 , 5 , 6^As of 1940, the practice of blood transfusions became widespread throughout Latin America, which contributed of the risk of CD by blood transfusion.^7 - 12^In this light, blood transfusions began to take on epidemiological importance as of 1944 and became technically approved in 1951. In 1969, the obligation of serological triage for CD was instituted in blood banks in Brazil. This action was established by the Brazilian Health Ministry in an attempt to control and increase safety against related diseases.^13^

Governmental decree number 158, ratified on February 04, 2016, by the Health Ministry, considers an ineligible donor to be those who have had home contact with the triatominae in an endemic area, and those with a clinical or laboratorial diagnosis for CD. In addition, in Article 130 of the same governmental decree, the conducting of serological tests of high sensitivity for CD became mandatory in every blood donation.^14^

Therefore, the present study sought to estimate the prevalence of *T. cruzi* in blood bank donors in the state of Ceará, given that the region has scarcely been explored. The results of the present study will serve as a warning to the epidemiological surveillance agency teams, so that they may establish measures for the prevention, treatment, and follow-up of individuals infected by *Trypanosoma cruzi* as well as for individuals who live in high-risk areas.

## Metodology

### Study design, location, and sample

This work is a retrospective descriptive study conducted through the collection of data recorded in the information technology (IT) system of the Hematology and Hemotherapy Center of the state of Ceará (HEMOCE in Portuguese) of all of the potential blood donors of the state’s Public Blood Bank Network from 2010 to 2015 ( Figure 1 ). The donors’ personal data were preserved, and these were identified by their registration number to guarantee confidentiality.

**Figure 1:** – Maps showing the location of the study. Source: Drafted by the author.

The state’s Public Blood Bank Network consists of a Blood Bank Coordinator, with its headquarters located in the city of Fortaleza; four Regional Blood Banks, located in the municipalities of Sobral, Quixadá, Crato, and Iguatu; one Blood Bank Nucleus, in Juazeiro do Norte; one Blood Bank Collection Clinic at the Dr. Jose Frota Institute (IJF in Portuguese); and 64 Blood Transfusion Agencies, located in the hospitals attended to by the Blood Bank Network of Fortaleza and the municipalities throughout the countryside of Ceará. Each blood bank is responsible for receiving blood donors and patients in the coverage area, with jurisdiction to conduct all of the blood cycle steps, with the exception of serology, which, for than a decade, has been centralized in the Blood Bank of Fortaleza, where the data referent to the blood donors was collected.

The serological triage for CD during the study period was performed by the chemiluminescence technique, which consists of the interaction of the antibodies present in the serum of the infected patients with antigenic epitopes present in the plaque, which, after incubation of biotinylated anti-gamma globin and streptoavidin conjugated with the enzyme, in the presence of a luminal reagent, are capable of emitting light.^15 , 16^

If the result is positive or inconclusive, the test is repeated in duplicate in the same sample. If the result remains positive or inconclusive, the patient is called to the blood bank service for a second blood collection. After the collection, the triage test (chemiluminescence) is again performed, and, if the result is positive or inconclusive in the repetition of the test, a confirmatory test will be performed and the donor is already definitively refused, even if the confirmatory test is negative. The confirmatory test must be a highly specific test, with the HEMOCE using indirect immunofluorescence (IIF) or *western blot,* ^15^depending on the bidding processes, since this is a governmental agency.

The IIF consists of a detection of antibodies in the infected serum, which, when incubated upon a slide with fixed *T. cruzi* antigens, connect themselves, forming the antigen-antibody compound, and which are revealed by a human anti-gamma globulin marked with fluorescein isothiocyanate. The *western blotting* test consists of a method in molecular biology and biochemistry to detect proteins in a homogenate or a biological tissue extract. This technique uses a gel electrophoresis to separate the denatured proteins by mass. The proteins are then transferred from the gel to a nitrocellulose membrane, where they are used as specific protein antibody probes.^15^

If the result is positive or inconclusive in the confirmatory test, the patient is referred to the medical clinic of the Walter Cantídio University Hospital or to Messejana Hospital, which are reference units for the clinical follow-up of individuals with chronic CD in the state of Ceará ( Figure 2 ).

**Figure 2:** – Fluxogram of the triage of potential blood donors for CD at the state´s Public Blood Bank in 2017. IIF: indirect immunofluorescence. Source: Drafted by the author.

### Data analysis

The occurrence of ineligible donors due to CD was calculated, and a descriptive statistical analysis was conducted from the absolute and relative frequencies. For the epidemiological characterization of individuals who are blocked from donating blood due to a confirmed diagnosis of CD, the following parameters were considered: gender (male and female), age range (18 – 29 years of age and over 30 years of age), profession, hometown, and city from which the individual came (Fortaleza or other municipality). The descriptive analysis was performed using Microsoft Excel, version 2013.

### Ethical aspects

The present study was approved by the Research Ethics Committee of the Federal University of Ceará (COMEPE-UFC in Portuguese), logged under protocol number 1.482.674, CAAE: 53833816.5.0000.5054, which was judged according to the guidelines for research with human beings from the National Health Council (Resolution CNS 466/12).

## Results

During the period of the study (January 2010 to December 2015), the state’s Public Blood Bank Network of the state of Ceará received a total of 763,731 potential blood donors. Of these, 155,378 (20.3%) were excluded in the clinical triage, and among the reasons for exclusion were: anemia, hypertension, alcoholism, drug use, high-risk behavior for sexually transmitted diseases, hepatitis, malaria, CD, among other causes. This study rejected 38 (0.02%) potential donors, of which 21 (55.2%) were men and 17 (44.8%) were women. Hence, the research on antibodies against *T. cruzi* (Serological triage) was conducted with 608,353 (79.6%) individuals.

During the period of the study, 14,159 blood bags were discarded, because they were believed to be inappropriate for donation due to positive serology for some type of disease traced at the blood bank, representing 2.32% of the total number of blood bags collected during the period. Of the 608.353 blood donor candidates approved in the clinical triage, 1,982 (0.32%) were considered ineligible for blood donation due to positive/inconclusive serology due to CD by means of the chemiluminescence test. Of these, 602 (30.37%) were positive and 1,380 (69.62%) were inconclusive ( Table 1 ). The inconclusive results corresponded to 9.75% (1,380) of the discarded bags.

**Table 1:** Total number of ineligible donors in the serology triage due to CD at the state´s Public Blood Bank from 2010 to 2015

YEAR	DONORS*	POS (N)	POS (%)	IND (N)	IND (%)	TOTAL INELIGIBLE (N)	TOTAL INELIGIBLE (%)
2010	94,847	86	0.09	244	0.25	330	0.34
2011	100,019	89	0.09	231	0.24	320	0.33
2012	98,815	89	0.09	374	0.38	463	0.47
2013	99,037	114	0.12	365	0.37	479	0.49
2014	105,281	112	0.11	84	0.08	196	0.19
2015	110,354	112	0.1	82	0.07	194	0.17
**TOTAL**	**608,353**	**602**	**0.1**	**1380**	**0.23**	**1982**	**0.33**

POS: positives; IND: undetermined. *Total number of donors who did the serology test. Source: Ceará Public Blood Bank (2010 – 2015).

The results showed an increase of 16.35% in the number of potential donors, when compared to 2010 to 2015. As regards the prevalence per year, a reduction of 50% (0.34% in 2010, 0.17% in 2015) was observed.

To verify if CD is still present in historically endemic areas, the prevalence was analyzed in the blood banks of Sobral, Quixadá, and Iguatu, considering positive and inconclusive results. The blood bank of Sobral presented the highest prevalence for CD in 2010 at 0.57% (78/13,674). In 2011, blood bank of Quixadá also presented 0.55% (29/5312), and in 2012 this same blood bank presented 0.71% (43/6,075), and 0.56% (33/5,914) in 2013 ( Figure 3 ).

**Figure 3:** – Prevalence of CD in the state´s Public Blood Banks, from 2010 to 2015. Source: Drafted by the author.

The blood banks of Quixadá, Sobral, and Iguatu presented the highest indices of serum prevalence for infection by *T. cruzi* in the period studied – 0.51% (171/33,413), 0.42% (356/85,150), and 0.40% (140/35,286), respectively.

The years of 2012 and 2013 presented the highest percentages of blood donors declared ineligible due to CD, 0.47% and 0.49%, respectively, with a subsequent reduction in the following years of 2014 and 2015, with the lowest values recorded of 0.19% and 0.17% ( Table 1 ). Of the ineligible donors due to CD, 847 (42.73%) had already donated blood some time before the period of this study.

Of the 1,982 donors selected to repeat the test (positive or inconclusive diagnosis in the serological triage due to CD), 757 (38.19%) individuals did not return to the Blood Bank to repeat the test. Hence, 1,225 (61.8%) individuals repeated the test.

In the repetition of the chemiluminescent triage test, 92 individuals presented inconclusive results, while and 333 presented positive results. These sample reagents were submitted to the confirmatory test (IIF or *western blot* ). Of these, 305 individuals presented a negative confirmatory test, 48 an inconclusive result, and 72 a positive result ( Table 2 ). Thus, 120 (28.23%) individuals were declared ineligible for blood donation due to CD.

**Table 2:** – Blood donors considered ineligible in the serology triage and recruited to repreat the test and take the confirmatory test from 2010 to 2015 (n: 1982)

REPETITION OF THE TRIAGE TEST (chemiluminescence)						CONFIRMATORY TEST (IIF ou Western Blot)			
YEAR	DID NOT RETURN	REPETITION NEG	REPETITION IND	REPETITION POS	TOTAL	CONF. NEG	CONF. IND	CONF. POS	TOTAL
2010	103	142	11	26	282	21	8	10	39
2011	124	126	13	47	310	49	11	11	71
2012	145	131	21	101	398	102	10	9	121
2013	160	235	18	54	467	41	1	6	48
2014	105	80	20	39	244	45	7	19	71
2015	120	86	9	66	281	47	11	17	75
**TOTAL**	**757**	**800**	**92**	**333**	**1.982**	**305**	**48**	**72**	**425**

*NEG: negativo; IND: indeterminado; POS: positivo; CONF: confirmatório. IIF: indirect immunofluorescence. Fonte: Hemorrede Pública Estadual do Ceará (2010 – 2015).*

Of these ineligible individuals due to CD (n=120), 78 (65%) were male, 100 were over 30 years of age, 100 (83.3%) were first-time donors, and 20 (16.7%) had already donated blood before. Forty-five (37.5%) of these individuals had completed high school and 32 (26.7%) had not completed elementary school, while 88 (73.3%) came from municipalities in the countryside of the state of Ceará and 62 (51.7%) came from the capital city of Fortaleza ( Table 3 ).

**Table 3:** – Sociodemographic characteristics of the donors considered ineligible with confirmation of CD at the Ceará Public Blood Bank from 2010 to 2015

	Positives		Undetermined		Total ineligible	
Variables	n	%	n	%	n	%
**Sex**						
Male	43	59.7	35	73	78	65
Female	29	40.3	13	27	42	35
Total	72	100	48	100	120	100
**Age**	**n**	**%**	**n**	**%**	**n**	**%**
≤ 30 years	8	11.1	12	25	20	16.7
>30 years	64	88.9	36	75	100	83.3
Total	72	100	48	100	120	100
**N of prior donations**	**n**	**%**	**n**	**%**	**n**	**%**
0	69	95.8	31	64.6	100	83.3
≥ 1	3	4.2	17	35.4	20	16.7
Total	72	100	48	100	120	100
**Education**	**n**	**%**	**n**	**%**	**n**	**%**
Illiterature	4	5.5	1	2.1	5	4.2
Elem. Incomplete	21	29.2	11	23	32	26.7
Elem. Complete	8	11.1	3	6.2	11	9.2
High School Incomplete	10	13.9	1	2.1	11	9.2
High School Complete	24	33.3	21	43.7	45	37.5
University Incomplete	0	0	5	10.4	5	4.2
Univeristy Complete	2	2.8	5	10.4	7	5.8
Not informed	3	4.2	1	2.1	4	3.3
Total	72	100	48	100	120	100
**Place of origin**	**n**	**%**	**n**	**%**	**n**	**%**
Fortaleza	7	9.7	15	31.2	22	18.3
Metropolitan region	3	4.2	4	8.3	7	5.8
Other municipalities	61	84.7	27	56.3	88	73.3
NI	1	1.4	2	4.2	3	2.5
Total	72	100	48	100	120	100
**City where they came from**	**n**	**%**	**n**	**%**	**n**	**%**
Fortaleza	30	41.7	32	66.6	62	51.7
Metropolitan region	16	22.2	6	12.5	22	18.3
Other municipalities	25	34.7	10	21	35	29.2
NI	1	1.4	0	0	1	0.8
Total	72	100	48	100	120	100

*Elem.: elementary; NI: not informed. Source: Ceará Public Blood Bank (2010 – 2015).*

## Discussion

Chagas disease, little by little, is becoming less relevant than it used to be in the realm of Brazilian sanitation. Even so, it still represents a challenge in some key aspects. Fiusa-Lima & Silveira in 1984^16^found an overall prevalence of 3.05% of CD infection in the northeastrne region (Brazil = 4.40%). Alencar conducted the first studies on CD in Ceará in 1987 and detected an estimated prevalence of 14.8%, highlighting the municipality of Limoeiro do Norte which presented 16.7%.^17^Despite the significant reduction in the vectorial transmission as of the 1990s, CD is considered by the World Health Organization (WHO) as “neglected”, which makes it part of a political and planned agenda of control, which should be followed by endemic countries.^18^

From the point of view of blood banks, the disease continues to be the object of continuous surveillance, given that the population of donors encompasses a considerable number of individuals who have already lived in sanitary and environmental conditions that were favorable to the transmission of the disease, and some of these individuals still live in these conditions. Thus, epidemiological studies in hemotherapy services becomes important, not only because of the relevance in blood transfusions, but also as an option to evaluate the transmission of the disease in specific municipalities.

In the present study, the prevalence of CD was of 0.33% in the serological triage of the Public Blood Bank Network of the state of Ceará. Similar results were also found in other regions of the country, such as in the study conducted at the Blood Bank of the city of Ituiutaba, in the Triângulo Mineiro region, which detected that 0.23% of the candidates presented a positive serology for CD, while 0.27% presented inconclusive serological results in the period of 2011 to 2011.^19^In the period from 1995 to 2009, a study was conducted in the Blood Bank of the Center Region of Uberaba (HRU in Portuguese) and a serum prevalence of 0.2% of ineligible donors due to CD was found.^20^

A bibliographic review conducted by Costa et al.^21^analyzed the ineligibility of candidates for blood donation due to seropositivity for CD in different regions of Brazil (northeast, southeast, south). In this sense, the greater prevalence of the ineligible donors due to seropositivity for CD occurred in the Northeast, specifically in the sity of Iguatu, CE, while in the southeast region, in the city of Patos de Minas, MG, the recorded percentages were of 1.90% and 1.20%, respectively. In the southern region of the country, the percentages of prevalence for ineligible donors due to seropositivity for CD varied between 0.40% and 0.47% in Porto Alegre and Pelotas, respectively.

The present study observed that the blood bank of Iguatu presented one of the highest prevalence of the studied period (0.41%); however, when compared to the findings from Costa et al.^21^a reduction was observed in the prevalence of ineligible donors due to seropositivity for CD in Iguatu, CE, between 2010 and 2015.

The blood bank of Sobral presented one of the highest prevalence of the studied period when compared to the other blood banks of the state ( Figure 3 ). These data can be explained by the fact that the city of Sobral is considered a high-risk region for vectorial CD. This municipality presents a unique eco-epidemiological scenario, characterized by foci of triatominae in informal urban settlements with low-quality housing. Species of native triatominae, such as *Triatoma brasiliensis* and *Triatoma pseudomaculata* can often be seen inside of houses, and are often infected with *T. cruzi* . These foci have the potential to develop acute cases of CD, illustrating that this region still represents a real concern for the population.^22^

Santana et al.^23^analyzed the positivity for CD among blood donors in Piauí between 2004 and 2013. The prevalence of the reagent serology for CD in the triage of blood donors was greater than that observed in the present study, at approximately 1%. Only 34.5% of the positive samples in the triage were referred for complementary tests. In the confirmatory tests, 84.4% presented negative results.^23^

In the state of Ceará, Silva et al.^24^studied the seroprevalence for CD in public blood banks from 1996 to 1997, where 34,943 donors were observed, of which 377 (1.1%) presented seropositivity for CD.^24^When compared to the present study, it could be observed that there was a reduction in the ineligibility of donors due to CD in the sorological triage that, until 2015, could be observed in 0.33% ( Table 1 ). This outcome possibly represents a reflection of the measures taken to improve the quality of hemotherapy services since the 1980s in many Brazilian states, which gave priority to the practice of return blood donations with fidelity and volunteer donors. Moreover, as the transmission in the rural areas has been diminishing due to anti-vectorial measures, it was expected that, over time, this new population of donors would present lower rates of infection.^24^

In addition to these factors, it should be highlighted that the techniques for serology have greatly progressed, which can be contributed to the reduction in the false positive results.^25 - 27^

In this context, the disease control policies must continue to exist, so as to avoid sporadic outbreaks, such as that which occurred in 1998 in the state of Ceará, due to the difficulties in continuing the anti-vectorial program (lack of insecticide and qualified professionals), which provoked a spike in the number of captured vectors and high frequency of reagent serology among the donors.^28^

One should be aware of the fact that the application of insecticides in homes does not seem to hinder the continuous reintroduction of wild triatominae species, and, as is well-known, the *Trypanosoma cruzi* circulates within a domestic zoonotic cycle, representing a challenge for the authorities involved in CD control. Concentrating efforts of sanitary surveillance in the three realms of government is necessary to eradicate this disease and its transmission via blood transfusion.^29 , 30^

It should also be emphasized that the hemotherapy services are not obligated by law to performed confirmatory tests for any of the diseases detected by routine exams. However, as set forth in Resolution RDC number 343, passed on December 13, 2002, as in other regulations, any donor presenting a non-negative serological result should be called to receive the necessary medical advice.^31^

The present study verified that many donors did not return for the repetition of the test, and this fact represents a limitation of the present study, as many potential donors did not perform the confirmatory tests. The non-return of the potential donors represents a loss of information, such as the more precise prevalence of CD in blood banks.

The blood bank network should formulate new strategies to recruit these individuals so as to confirm the diagnosis and, in the case of a negative result, allow them to become blood donors once again.^31^

This loss of information is worrisome and can result from a combination of factors, among which is the place of origin of many donors. In addition to the difficulty of the donors travel from his/her city of origin to the capital, the letter of summons is an instrument that scares people, as it indicates that the result of the exam was not normal, and many end up not wanting to know the true result.^15^

The present study observed that the majority of ineligible donors due to CD (positive/inconclusive) was of the male sex, over 30 years of age, from the capital city of Fortaleza, but born in the municipalities in the countryside of the state of Ceará. Although other studies also show this predominance of males in individuals diagnosed with CD, there is no positive correlation between the sex of the donor and the reagent serology for the disease, as it indistinctly affects both men and women, which can be justified by the fact that many men work in rural areas, with greater chances of coming into contact with the triatominae, as well as due to cultural differences in the practice of blood donations, which commonly attribute to the best donor role to the men.^28 , 32 - 34^

As regards the place of origin, it is well-known that many knowingly infected individuals live or have lived in countryside regions that constitute the natural ecotopes of the insect vector and, later, many end up migrating to large urban centers, such as Fortaleza, fleeing from droughts and in search of jobs and opportunities.^35^Despite the certificate of the eradication of CD by *Triatoma infestans* , it is important to clarify that this species was never found in the state of Ceará; therefore the risk of transmission of CD in Ceará is due to the most prevalent species of triatominae, the *Triatoma brasiliensis* and the *Triatoma pseudomaculata.* Thus, the state of Ceará still presents a high risk for the transmission of the disease.^3 , 35 - 37^

In addition to the vectorial form, the form of transmission has been especially attacking the northern and northeastern regions of the country. In the case of men, this transmission can occur in a sporadic manner, through the ingestion of food contaminated with the parasite or its feces.^38^

As regards the age range, during the period of study, a reduction in the number of younger infected individuals could be observed, which can be evaluated as a reflection of the vectorial control measures implemented in the state of Ceará. With the overall reduction of the incidence of the disease, the entrance of individuals diagnosed with CD became less frequent in the age group of people who donate blood, parallel to the progressive exit of infected individuals from the list of donors by age or by morbidity due to CD. However, it is important to highlight that the increase in the launching of blood donation campaigns generates a greater demand for people of highly varied age groups, making it difficult to identify a greater difference between the analyzed age ranges.^39 , 40^

Parallel to the reduction in blood donors diagnosed with CD in Ceará, what calls attention is the high proportion of inconclusive reactions. During the period of the study (2010-2015), we observed that, in the serological triage, 70.9% (1,380) of the results of ineligible donors due to CD were inconclusive, representing 5.99% of the total number of bags discarded during the period. Many studies demonstrate that the inconclusive reactions often represent more than 50% of the cases of donor ineligibility due to seropositity for CD in blood banks, with this rate being higher than 70% in some services.^41 , 42^

In Brazil, it is estimated that 60% of the occurrences of inconclusive reactions in the three million annual blood donations, of which 0.6% of the collected blood bags are discarded due to a positive serology for *T. cruzi* , that is, 10,800 bags are being discarded due to inconclusive serology.^34^The significant number of inconclusive reactions is worrisome, especially since, in addition to the high costs caused by the disposal of blood bags, there is also the consequence that this brings to the donor, who is labeled as an individual diagnosed with a severe disease such as CD.

Discrepancies in the results of the serological tests occur quite often, sometimes questionable in a given text and positive or negative in another. The present study verified that 847 individuals had previously donated blood and such discrepancies become even more evident and conflicting in repeat donors, when more than a dozen repeatedly negative serological reactions in previous donations now present an inconclusive or eventually positive serology in a subsequent donation.^43 - 45^

The present study verified that of the 1,982 ineligible donors due to CD, 1,225 (61.8%) returned to repeat the test, and of these, only 72 (5.8%) were positive in the confirmatory test (IIF or *western blot* ) and 48 (3.9%) were inconclusive. The occurrence of inconclusive reactions and false-positive reactions in the tests of serological triage show flaws in the specificity of the serological tests, as there may be many individuals co-infected with other diseases, and therefore sensitized with other antigens, which can entail crossed reaction in the serological tests.

Consequently, many healthy individuals end up being labeled as CD carriers, when in fact they are not actually infected with the disease, leading to psychological, social, and economic issues for the donor who was excluded for wrongly being considered infected with CD, in addition to promoting the unnecessary disposal of blood bags in blood banks and important financial losses for the Brazilian Unified Health System.^40^

The data obtained in this study reflect the difficulties in the approach and guidance of donors with inconclusive serological reactions, who are almost all non-chagasic, as well as the indispensability of the implementation of measures that allow one to minimize, or even eliminate, the questionable or false-positive serological results for CD in serological triage tests.

The confirmatory tests present good sensitivity and specificity; however, the triage tests, such as chemiluminescence, present a high sensitivity, and it is well-known that tests with a high sensitivity have their specificities compromised, generating false-positive results. Due to this factor, it is important to implement more specific tests in the serological triage of blood donors.^28 , 30 , 31^

The determination of the prevalence of the disease in blood banks can be relevant as an indicator of the risk for CD in blood transfusions and of the level of transmission of the disease in a determined region, as well as allow one to evaluate the vectorial control indirectly, providing up-to-date information about the disease in the state.

In this sense, it is possible to trace local strategies that involve the efforts of all of the sectors related to the area, such as sanitation agencies, blood banks, and laboratories, unified in the effort to eliminate the transmission through blood transfusions and improve the quality of the transfused blood.

In addition, the results from this study warn of the need in the blood banks to introduce a complementary serological method that is more specific, in an attempt to minimize the unnecessary disposal of blood bags and consequently indicate the real values of the disease in blood donors. Hence, there is a need for studies that propose new measures for the improvement in the accuracy of the serological tests, which could consequently reduce the unnecessary disposal of blood bags, thus diminishing the costs for the Brazilian Unified Health System.

## Conclusions

Of the potential donors in the studied period, 1,982 were considered ineligible donors due to a positive/inconclusive serology for CD. Confirmed as ineligible (positive or inconclusive) were 28.2% (120/425) due to CD. No significant reduction in positive/inconclusive serology was observed in the period between 2010 and 2015; however, a reduction was observed in relation to 1996/1997 in the state. The determination of the prevalence of CD in blood banks can be relevant as an indicator of risk for transmission through blood transfusion in a given region. New serology tests for triage, with a better accuracy, are necessary in order to reduce the unnecessary disposal of blood bags, reduce the costs for the Brazilian Unified Health System, and diminish the insecurity for patients and family members.
